# Supplementary material for: The Pain at Work Toolkit for Employees with Chronic or Persistent Pain: A Collaborative-Participatory Study
Source: Healthcare (Basel). 2021 Dec 29;10(1):56. doi: 10.3390/healthcare10010056 (PMC8775489; doi:10.3390/healthcare10010056)
Supplement: Supplementary file 1 [file healthcare-1520298-File S2. Expert peer review.pdf › healthcare-1520298-File S2. Expert peer review.pdf]

Supplementary file S2. Expert peer review

Reviewers were asked to select a yes/no response for each item, and then expand on their answer if they had further comment. Reviewers were asked whether the focus of the resource was clear and consistent, whether the information was factually correct, and whether the text was well written and in clear sentences. They were asked whether the resources links signposted them to the required information and whether the broad sections of the package were appropriate. Reviewers were then asked to comment on the overall appropriateness of the package with regards layout, images and links. They were asked to comment on the ease or difficulty of initial access to the package via the web link, and whether or not the package could be accessed in different settings (e.g., work or home). Finally, they were asked about the relevance of the package to people with chronic or persistent pain. Questions items are shown in the Table below.

Professionals Review Question Items
1. Is revision to the toolkit required?
2. Is the focus of the resource clear and consistent?
3. To your knowledge is the information factually correct?
4. Is the text well-written and in short, clear sentences?
5. Do the suggested links provide the information needed? Are there any other links to additional resources that you could suggest?
6. Are the broad sections appropriate?
7. Is the overall presentation appropriate? (layout, image links)
8. How easy is this resource to access via the link?
9. Could this be accessed in different settings? (workplace, home)
10. Is this toolkit relevant to any employee who has chronic or persistent pain?
